# The Paroxysmal Depolarization Shift: Reconsidering Its Role in Epilepsy, Epileptogenesis and Beyond

**DOI:** 10.3390/ijms20030577

**Published:** 2019-01-29

**Authors:** Helmut Kubista, Stefan Boehm, Matej Hotka

**Affiliations:** Center of Physiology and Pharmacology, Department of Neurophysiology and Neuropharmacology, Medical University of Vienna, Waehringerstrasse 13a, 1090 Vienna, Austria; stefan.boehm@meduniwien.ac.at (S.B.); matej.hotka@meduniwien.ac.at (M.H.)

**Keywords:** hippocampal neurons, L-type voltage-gated calcium channels, seizures, Alzheimer’s disease, electrophysiology, neuronal dysfunction, giant depolarizing potentials, neuronal remodelling, dendrites

## Abstract

Paroxysmal depolarization shifts (PDS) have been described by epileptologists for the first time several decades ago, but controversy still exists to date regarding their role in epilepsy. In addition to the initial view of a lack of such a role, seemingly opposing hypotheses on epileptogenic and anti-ictogenic effects of PDS have emerged. Hence, PDS may provide novel targets for epilepsy therapy. Evidence for the roles of PDS has often been obtained from investigations of the multi-unit correlate of PDS, an electrographic spike termed “interictal” because of its occurrence during seizure-free periods of epilepsy patients. Meanwhile, interictal spikes have been found to be associated with neuronal diseases other than epilepsy, e.g., Alzheimer’s disease, which may indicate a broader implication of PDS in neuropathologies. In this article, we give an introduction to PDS and review evidence that links PDS to pro- as well as anti-epileptic mechanisms, and to other types of neuronal dysfunction. The perturbation of neuronal membrane voltage and of intracellular Ca^2+^ that comes with PDS offers many conceivable pathomechanisms of neuronal dysfunction. Out of these, the operation of L-type voltage-gated calcium channels, which play a major role in coupling excitation to long-lasting neuronal changes, is addressed in detail.

## 1. How can a Paroxysmal Depolarization Shift (PDS) Be Defined?

**Figure 1:**
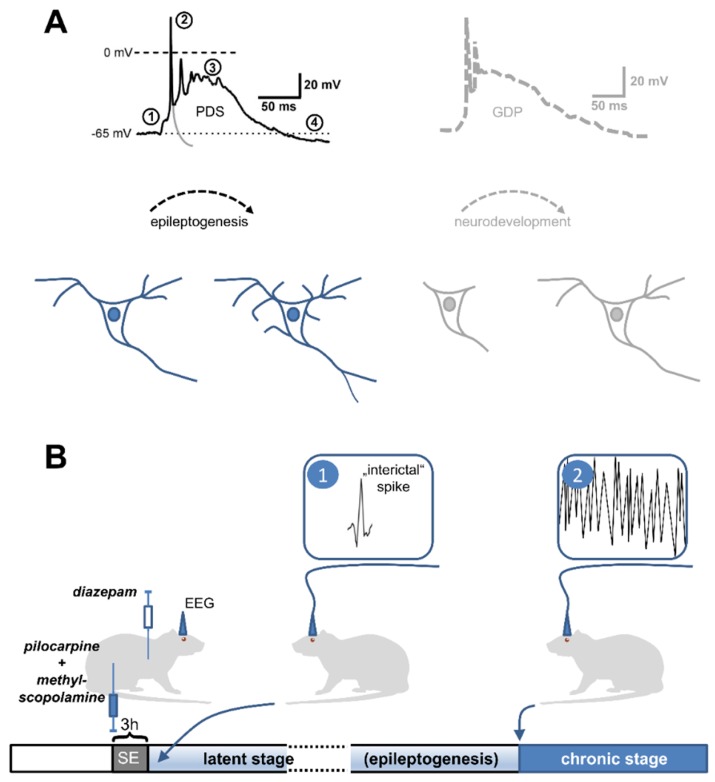
Observations in favor of an epileptogenic role of PDS. (**A**) Resemblance of PDS to giant depolarizing potentials (GDPs). The black trace depicts a typical PDS, which is synaptically triggered (1) and consists of action potentials of decreasing amplitude (2), a depolarized plateau (3) and termination by repolarization, sometimes below the resting membrane potential (after-hyperpolarization, (4) (to highlight the difference between a single action potential and a PDS, a hypothetical repolarization trajectory of the initial action potential is indicated by the grey line). This appearance is reminiscent of GPDs (an example is retraced in grey on the right side from a paper by Ben-Ari et al., 1989 [89]), which are widely believed to govern neuronal development [90]. In analogy, PDS may initiate various, potentially pathogenic neuronal changes. Neurodevelopmental (bottom right) and neuropathological morphological changes (bottom left) are indicated in schemes of a neuron below the traces. (**B**) Early appearance of electrographic spikes, the multi-unit correlate of PDS, in animal models of acquired epilepsy. Post-status epilepticus models are widely used in epilepsy research to investigate the mechanisms of epileptogenesis. In the pilocarpine version of this model [91], the cholinergic agonist pilocarpine is injected (together with non-brain-permeant methyl-scopolamine to avoid peripheral cholinergic side effects) into rats to evoke status epilepticus (SE). After 3 h, SE is terminated by the application of the benzodiazepine-type GABA_A_ receptor modulator diazepam. Continuous electroencephalographic recording (EEG) is performed to monitor the appearance of ictal discharges (2), which typically starts after days to weeks (chronic stage). The time until the first occurrence of seizures is referred to as the latent (or silent) stage. Electrographic spikes (1) were found to occur within 24 h after the insult (here SE), i.e., at the starting point of epileptogenesis (it should be noted that these electrographic spikes are commonly referred to as “interictal” in the literature, although the term “pre-epileptic” would be more appropriate to avoid confusion with pre-ictal or truly interictal spikes).

Paroxysmal depolarization shifts (PDS) are abnormal fluctuations of the neuronal membrane voltage. Although they are brief, PDS far outlast the depolarization of normal action potentials (Figure 1A). PDS typically show a distinct voltage trajectory, which consists of action potential discharge at the onset with progressive amplitude reduction until only small oscillations remain riding on top of a depolarized plateau. However, variability was demonstrated for the depolarized plateau, which amounts to about 20 to 50 mV, and for the PDS duration, which lasts from tens to several hundreds of milliseconds [1,2,3]. The first descriptions of PDS date back to experimental epilepsy research of the 1960s. Back then, PDS were identified in penicillin-treated foci of cat cortices as cellular correlates of (pre-ictal) electrographic events [1,2]. In these studies, PDS occurred spontaneously during penicillin-induced neuronal activity, but could not be triggered by electrical stimulation via intracellular recoding electrodes [2]. From our current understanding, the induction of PDS can be explained by inhibition of GABAergic inhibition, commonly known as disinhibition, as penicillin at high doses acts as a GABA_A_ receptor antagonist [4]. In line with this interpretation, PDS similar to those evoked by penicillin were detected in brain slices upon application of the GABA_A_ receptor antagonists picrotoxin [5], pentylenetetrazol [6], and bicuculline [7,8]. In addition, evidence was provided that Ca^2+^ influx via L-type voltage-gated calcium channels (LTCC) contributed to the appearance of typical PDS waveforms, whether evoked *in vivo* by penicillin or in brain slices by pentylenetetrazol and bicuculline, respectively [6,7,9,10].

Over the years, the term PDS has been used to describe a variety of different epileptiform discharges, including epileptic bursts, segments of seizure-like activity and post-ictal discharges [11,12,13,14]. However, these latter epileptiform events often lack characteristic features of original PDS, in particular in terms of event duration (for example up to 30 s long, see [15]) and/or spike amplitude reduction during the depolarized plateau. Moreover, evidence was provided that PDS have a dendritic origin [8], whereas epileptic bursts predominantly arise from a perisomatic region and can be evoked by depolarizing current injections into neuronal somata [16,17,18,19]. Notably, abnormal depolarizations lasting from seconds to minutes upon induction by epileptogenic drugs in invertebrate neurons are also referred to as PDS [20,21], although they share only marginal similarity with PDS of mammalian neurons. The broad use of the term “PDS” hampered respective research and blurred our current understanding of their role in epilepsy. In our opinion, PDS sui generis should be differentiated from various other electrical events that have been provided with the same name in a rather misleading manner. Hence, the current review deals with waveforms that closely resemble PDS as originally identified by Matsumoto and Ajmone Marsan, 1964 [1] and Prince, 1968 [2]. These early reports revealed that PDS were accompanied by electrographic events of similar duration that were mostly characterized as interictal spikes (IIS). Importantly, the precise pattern of PDS and their association with electrographic signals appears of crucial importance when considering their role in epilepsy and/or epileptogenesis (see below, Section 4). 

## 2. What Are the Mechanisms of PDS Formation?

Regarding the question as to how PDS may arise, two ideas have emerged initially: the “synaptic theory” held that excessive stimulation of otherwise healthy neurons, e.g., because of recurrent synaptic feedback, was responsible for the abnormal depolarizations. The “epileptic-neuron theory” posed that quasi healthy synaptic potentials led to PDS because of alterations in intrinsic properties of the affected neurons. Indeed, PDS-like events can be evoked by alterations in neurotransmission, as in the “relief from kynurenate + high Mg^2+^ model” [22] or the “GABA withdrawal model” [11,23]. Alternatively, PDS can also be evoked by changes in intrinsic neuronal properties such as calcium homeostasis (caffeine model [24]). In primary hippocampal networks (as well as in hippocampal slices, see references [7,25,26]), PDS can be readily evoked by inhibition of fast inhibitory neurotransmission using GABA_A_ receptor blockers. Bicuculline-induced PDS only occurred when fast excitatory neurotransmission was active, because addition of the α-amino-3-hydroxy-5-methyl-4-isoxazolepropionic acid (AMPA) receptor blocker 6-cyano-7-nitroquinoxaline-2,3-dione (CNQX) abolished PDS discharges [27]. This indicates that synaptic imbalance can be a precipitating cause of PDS formation. Nevertheless, the distinct voltage trajectory of PDS required the availability of LTCCs, as it was lost upon inhibition of LTCCs with the dihydropyridine-type LTCC blocker isradipine [27,28]. In the presence of isradipine, bicuculline-induced electrical events appeared as enhanced excitatory postsynaptic potentials (EPSPs), but did not correspond to classical PDS [1,2]. Hence, it is possible that both types of induction mechanisms may play a role; i.e., enhanced intrinsic conductances can lead to PDS even with normal synaptic activity, and PDS can also occur in neurons with normal intrinsic conductances when outsized EPSPs are formed. Both mechanisms are likely interdependent, as enhanced intrinsic conductances may only be triggered by synaptic input, whereas enhanced synaptic input may activate abnormal levels of per se unaltered voltage-dependent conductances. Notably, the work of Michael Segal in the early 1990s demonstrated that PDS can even be elicited in microcultures containing only one autaptic excitatory hippocampal neuron [29]. We have found in our own experiments that LTCC-mediated Ca^2+^ influx, which represents a somatodendritically-localized single-cell property, is crucial for PDS formation. Importantly, potentiated LTCC-mediated Ca^2+^-influx appears to stand at the core of endogenous factors that promote these abnormal electrical events (see Section 2.1), because in current injection-induced depolarizations in the presence of tetrodotoxin (when synaptic inputs and action potential discharge are disabled) the LTCC agonist Bay K8644 gave rise to PDS-like voltage responses (unpublished observations). Moreover, Speckmann et al., 1990 [30] had demonstrated in earlier work that PDS elicited in-vivo in the rat motor cortex were abolished by intracellular application of the verapamil (an LTCC-antagonist)-derivative D890, whereas application of the LTCC potentiator Bay K8644 led to their augmentation.

### 2.1. Which Ion Conductances Underlie PDS?

A crucial role of LTCCs, i.e., Ca_v_1.3 channels, in PDS was confirmed recently in our lab using genetically modified mice [27]. This study also provided evidence arguing against a contribution of Ca^2+^-dependent nonspecific cation (CAN) channels, which had been implicated from theoretical considerations previously [31]. In addition to LTCC-mediated Ca^2+^ influx, evidence has also been provided that *N*-methyl-d-aspartate (NMDA) receptor-mediated cation current contributes to PDS [32,33,34,35,36], although the effect of the NMDA receptor blocker AP-5 (d(−)-2-amino-5-phosphonopentanoic acid) on PDS appeared more pronounced in neocortical neurons than in hippocampal neurons [37,38]. However, it is difficult to determine the relative contribution of NMDA- and LTCC-mediated currents, since functional interplay was demonstrated between these two channel classes in neurons [39,40,41], e.g., NMDA receptors providing a critical excitatory component that may ultimately activate LTCCs [42]. Hence, blocking NMDA receptors may also eliminate a LTCC-dependent component.

Various inhibitory mechanisms have been proposed to contribute to termination of PDS, in particular K^+^ and Cl^−^ conductances as well as GABA_B_ receptor mediated signaling [43,44]. However, experimental evidence in support of the impact of one of these conductances on PDS termination remained sparse [45,46]. Controversial results have been reported regarding the contribution of GABA_B_ receptor-mediated mechanisms [43,45]. Moreover, depolarizing afterdischarges rather than inhibitory afterpotentials were also observed to follow PDS [5,33,36,38,47], which may be due to a masking of inhibitory mechanisms by depolarizing Cl^−^ currents through GABA_A_ receptors or NMDA receptor mediated currents [46,48]. Obviously, when GABA_A_ receptor blockers are used for PDS induction (i.e., to mimic GABAergic loss, see Section 2.2.), the relative role of non-GABA_A_ receptor-mediated processes will predominate in shaping PDS trajectories.

Two additional conductances should be considered when discussing the foundations of PDS. The two relevant ion channels have been implicated in epileptic bursts, discharge patterns that have been identified in the pilocarpine model of acquired epilepsy [16]. However, epileptic bursts appear days to weeks after the insult [19,49] rather than hours, as seen for PDS [50]. They rely on Na_v_ channels that give rise to a persistent sodium current (I_Na,p_), and on T-type voltage-gated calcium channels (TTCCs, Ca_v_3.* family) [17,18,19]; they are generated in the perisomatic region, and can be readily triggered by somatic current injection via a microelectrode. However, our experiments using riluzole at low micromolar concentrations, which are known to block I_Na,p_, provided evidence that I_Na,p_ is not involved in PDS [27]. With respect to TTCCs, it should be noted that PDS can also be induced in less polarized hippocampal neurons (e.g., V_m_ of about −55 to −50 mV) as observed in our own experiments in primary cultures (unpublished observation) and by others using isolated guinea pig brains [51]. Since TTCCs are largely inactivated at such subthreshold membrane potentials [52], they are highly unlikely to play a significant role in PDS.

Other conductances that have been named repeatedly in conjunction with PDS-like events are provided by transient receptor potential (TRP) channels. Phelan and co-workers described pronounced and long-lasting PDS-like “epileptiform bursts”, and showed that these events depended on an activation of canonical TRP (TRPC) channels via metabotropic glutamate receptors (mGluR) [53]. With respect to duration, these PDS-like events considerably exceeded those originally recorded in pre-seizure penicillin-induced foci [1]. However, this observation indicates that neuromodulatory alterations may enable additional conductances that either prolong PDS or eventually cause transition to seizure-like discharge activity, which may result from a melting of single PDS [14]. In support of this conclusion, a molecularly undefined, Ca^2+^-dependent nonspecific cation channel was demonstrated to cause runs of PDS, and only its inhibition by flufenamic acid turned these runs, which were classified as “seizure-like events”, into isolated PDS discharges [54]. 

In summary, the ionic basis of a PDS discharge can be described as follows (illustrated in reference [55]): the PDS emerges from a network-driven giant EPSP triggered by AMPA receptor-mediated fast neurotransmission. Its depolarizing plateau is mediated by NMDA receptor channel current and current through LTCCs, in particular Ca_v_1.3 channels. The action potentials are due to the activation of voltage-gated sodium channels, and the amplitude decline and/or firing cessation is likely due to progressive inactivation of these channels during the continuous depolarization. The termination of PDS may involve different ionic mechanisms depending on the expression pattern of various ion channels in a particular type of neuron; relevant ion channels are Ca^2+^-dependent (e.g., apamin sensitive-) K^+^ channels, ionotropic GABA_A_ receptors as well as metabotropic GABA_B_ receptor-regulated channels. I_Na,p_ and TTCCs do not seem to represent essential components of PDS. However, the presence of further ion channels in a particular type of neuron and/or their metabotropic activation may modify the voltage trajectory of individual PDS or the sequence of PDS discharges.

### 2.2. How Are the Mechanisms of PDS Formation Induced?

Insight into the mechanisms of PDS formation can be gained from their occurrence in experimental forms of acquired epilepsy. As will be discussed in more detail below, electrographic (“interictal”) spikes (IIS) have been demonstrated to occur as early as day one after the insult in post-status epilepticus (post-SE) models, whereas spontaneous unprovoked seizures occur after a latent period of up to a few weeks only [50] (Figure 1B). Hence, epileptogenic mechanisms that have been reported to occur early on (e.g., within 24 h after the incident) in post-SE models represent the most likely candidates responsible for the generation of interictal spikes and thus for their cellular correlate PDS. Such changes comprise (a) reduction of GABAergic neurotransmission (e.g., loss of interneurons) [56,57]; (b) augmentation of glutamatergic neurotransmission, for example through phosphorylation of GluR1 AMPA receptors with concomitant enhancement of AMPA receptor-mediated synaptic currents [58,59] or up-regulation of NMDA receptors [60] (together, the reduction of GABAergic and increase of glutamatergic neurotransmission may explain why EPSP cannot be controlled properly, thus enabling giant EPSPs that drive PDS); (c) disturbances of intracellular Ca^2+^-homeostasis [61,62]; (d) formation of reactive oxygen species [63,64]; and (e) elevations of intracellular chloride (e.g., by Na^+^ –K^+^ –Cl^−^ co-transporter1 (NKCC1) up- and K^+^ –Cl^−^ co-transporter 2 (KCC2) down-regulation) [65,66]. The reduction of GABAergic neurotransmission is mimicked by the most widespread and reliable experimental induction method of PDS, i.e., the inhibition of GABA_A_ receptors with high doses of penicillin or pentylenetetrazole [10,67,68,69] or micromolar concentrations of picrotoxin or bicuculline [5,7,8,25,54,67]. An up-regulation of glutamatergic neurotransmission was experimentally employed in the “relief from kynurenate + high Mg^2+^ model” to induce PDS discharge [22]. The caffeine-induction method of Moraidis et al., 1991 [24] can be envisaged to demonstrate that disturbances of the intracellular Ca^2+^-homeostasis at least in part may provoke PDS formation [28]. Likewise, hydrogen peroxide application [28] and direct elevation of intracellular chloride via a patch pipette (unpublished observations) both promoted the formation of PDS-like events, although less efficiently than GABA_A_ receptor antagonism.

The widely observed contribution of LTCCs to PDS may arise from LTCC augmentation via reactive oxygen species (see for example reference [70]) and promotion of their activation by enhanced excitatory synaptic input (e.g., to a voltage range where window currents via LTCCs occur, see reference [71]), or changes in cytosolic Ca^2+^ (Ca^2+^-dependent facilitation) [72]. However, there is only limited evidence for a role of any of these mechanisms in PDS formation to date. For example, caffeine did not induce PDS in Ca_v_1.3^−/−^ neurons [27], and hydrogen peroxide was most effective in PDS induction in neurons with a pronounced availability of LTCCs [28]. The implication in PDS formation of LTCC up-regulation by hormones associated with neuronal stress or injury, e.g., glucocorticoids [73] or insulin-like growth factor-1 [74], has also remained unexplored to date. Evidently, more work is required to investigate by which mechanisms LTCC activity may be potentiated to promote PDS discharge in a manner similar to the one demonstrated by pharmacological LTCC enhancement using Bay K8644 [27,28]. Interestingly enough, gain-of-function mutations of CACNA1D (which is the gene coding for Ca_v_1.3) but not of CACNA1C (coding for Ca_v_1.2) have been linked to epilepsy [75,76]. Hence, up-regulation of Ca_v_1.3 may not only play a role in acquired forms of epilepsy but may also be implicated in genetically determined predisposition to epilepsy.

## 3. Synchronization of PDS

Our current understanding of how PDS are synchronized is very limited. Several mechanisms have been proposed to account for neuronal synchronization during epileptiform discharges in general. These include electrical communication via intercellular gap junctions, electrical field effects (ephaptic interactions), decreased volume of the extracellular space (e.g., due to cell swelling), increased extracellular potassium and GABAergic depolarization resulting from increased intracellular chloride, but also synchronous recovery from inhibition of an assembly of principal neurons. [77,78]. Nevertheless, it remains unclear which of these mechanisms can contribute to the synchronization of those PDS that give rise to individual interictal spikes. As PDS appear to arise from the dendritic compartment, synchronization may occur in dendritic layers. Brain-derived neurotrophic factor (BDNF), for example, is secreted in an activity-dependent manner at dendritic sites [79], has glutamate-like fast excitatory and slow modulatory effects on synaptic plasticity [80,81] and is known to play a role in neuronal synchronization [82,83,84]. Hence, although there is no direct experimental evidence yet, PDS-induced release of neuromodulators from dendritic sites can also be envisaged to play a role in PDS synchronization.

## 4. Role in Epilepsy/Epileptogenesis

PDS have been monitored both as individually occurring electrical events as well as closely spaced in clusters [1,2,5,8]. This is mirrored by interictal spikes, which were shown to occur in a sporadic manner or in the form of brief paroxysmal bursts in animal models of epilepsy [85]. As will be discussed in Section 4.2., the frequency of interictal spikes (and therefore of cellular PDS) prior to or after ictal discharges has inspired hypotheses regarding their potential roles in epilepsy. Some authors claimed that ictal discharges can be regarded as a melting of PDS [14], with individual PDS representing the “hallmark” of epileptic discharges [44]. However, due to differences in EEG patterns, others doubt that ictal discharges may be seen as reinforcement and/or acceleration of discharges that underlie interictal spiking [86].

Indeed, considering the inactivation/deactivation properties of various ion conductances that contribute to PDS (see Section 2), increased PDS frequency can be envisaged to come with pronounced changes in the ionic composition of the resulting seizure-like discharges [55]. Hence, PDS may play a neuropathogenic role distinct from that of ictal discharges.

The strong association of IIS with epilepsy has been widely utilized for diagnostic purposes. However, no consensus has been reached regarding the implication of IIS in epilepsy. Initially, IIS were largely considered as an insignificant by-product of the epileptic condition, but in the last decades hypotheses on pro-epileptic (i.e., epileptogenic) and anti-epileptic (i.e., anti-ictogenic) roles have emerged [87,88]. These opposing ideas on the roles of IIS will be discussed in the following Section 4.1 and Section 4.2. We will consider both, evidence from studies on IIS and on PDS, and will therefore use the term “IIS/PDS”, unless information given refers only to the extracellularly detected events (IIS) or events that were recorded solely with intracellular methods (PDS).

### 4.1. An Epileptogenic Role of PDS

The hypothesis of a pro-epileptic role of IIS/PDS emerged from two considerations originally disseminated in 2005 by Staley and his colleagues [87]: first, PDS are more or less identical to giant depolarizing potentials (GDPs), which occur in a synchronous manner in neurons of neonatal rats up to an age of about two weeks [89] and which are widely believed to play a crucial role in neurodevelopment [89,92]. GDPs are driven by excitatory GABA_A_ receptor-dependent neurotransmission and involve Ca^2+^-influx via voltage-gated calcium channels (putatively LTCCs) and NMDA receptors [93,94]. As described above, LTCCs and NMDA receptors play a crucial role in PDS formation as well. The L-type channel family was reported to have a privileged role among voltage-gated calcium channels in excitation-transcription coupling [95,96]. Notably, mitochondrial production of reactive oxygen species (ROS), which is augmented during the latent period of epileptogenesis [64] (see Section 2.2.), was shown to augment the activity of the LTCC-regulated gene transcription factor CREB (cAMP-responsive element binding protein) [97]. Hence, PDS may promote long-term morphological as well as functional (synaptic plasticity) changes [98]. In line with such a role of LTCC-mediated Ca^2+^ influx during PDS, IIS were found to drive activity-dependent gene expression [99]. The similarity between GDPs and PDS raises the possibility that PDS were capable of initiating a recapitulation of developmental processes in fully matured neuronal circuits (Figure 1A). This may eventually lead to the formation of hyperexcitable circuits.

Secondly, spontaneous unprovoked seizures occur only after a latent period in both, the post-SE model and the kindling model of acquired epilepsy, but IIS can be monitored much earlier [50,100,101,102,103], e.g., as early as day one after the insult in post-SE models [50] (Figure 1B). Likewise, in humans who acquired epilepsy as a result of war-related brain insults, IIS preceded the first seizures [87,104]. Moreover, in two epileptic rat models (i.e., SER, spontaneously epileptic rats, and NER, Noda epileptic rats), PDS were identified in CA3 hippocampal neurons [105,106], and their occurrence was reported to precede the first spontaneous seizures that appear in these animals at several weeks to months of age [106]. Taking both considerations together, i.e., the similarity of GDPs and PDS on the one hand, and the early appearance of IIS in epileptogenesis on the other hand, it can be hypothesized that PDS may not simply represent a by-product of a developing epileptic condition in the brain, but may actually play a key role in the epileptogenic processes.

As indicated above, LTCC-mediated Ca^2+^-influx was suggested to be a crucial step in CREB-dependent gene regulation by excitatory, GDP precipitating GABA activity in neurodevelopment, which leads—for instance—to an increased expression of BDNF [107]. Analogous mechanisms can be expected to play a role in epileptogenesis. Notably, over-expression of BDNF led to the formation of basal dendrites and to axonal branching in dentate granule cells in hippocampal cultures [108]. Excessive activation of LTCCs by stimulating the dentate gyrus with picrotoxin was shown to induce the expression of BDNF in granule cells which via TrkB receptors causes axonal branching in mossy fibers [109]. Picrotoxin also induced the formation of recurrent excitatory inputs to granule cells in an LTCC-dependent manner, but picrotoxin-induced sprouting in mossy fibers was independent of LTCCs [110]. Other authors demonstrated that BDNF induced neurogenesis and led to the formation of ectopic hippocampal granule cells [111]. Notably, ectopic granule cells were also seen in the pilocarpine or kainate models of epilepsy [112,113,114]. In line with an epileptogenic role of such BDNF-mediated changes, limbic kindling was shown to cause aberrant neurogenesis and basal dendrite formation together with increased BDNF expression [115]. Moreover, in animal models of acquired epilepsy, increased neurogenesis and morphological changes of granule cells (aberrant dendritic projections, e.g., hilar basal dendrites, aberrant axonal sprouting, which creates recurrent excitatory loops [116]) occur in the dentate gyrus of the hippocampus after an epileptogenic insult [117]. These changes occur early during epileptogenesis, i.e., within one day [118], and the dentate gyrus shows pronounced expression of LTCCs [119,120,121]. Ca_v_1.3 channel-mediated activation of CREB was demonstrated in hippocampal neurons [74,122] and appears to be distinct from the one mediated by Ca_v_1.2 channels [122]. The preferential role of Ca_v_1.3 in PDS formation is intriguing, the more so because this channel has been identified as a risk gene in human epilepsy, and up-regulation of Ca_v_1.3 (together with Ca_v_1.2) has been also demonstrated in animal models of acquired epilepsy [123]. Taken together, circumstantial evidence points to CREB-dependent gene transcription, BDNF expression and release, neurogenesis, as well as dendritic and axonal remodelling as epileptogenic sequelae of PDS (illustrated in reference [55]), even though directly supporting experimental evidence is lacking.

According to the above-mentioned epileptogenic role of PDS, LTCC inhibitors should counteract epileptogenesis. This possibility has been addressed rarely, although—for example—Mikati and colleagues found that the dihydropyridine-type LTCC antagonist nimodipine significantly ameliorated the outcome in the kainate-induced SE model of epileptogenesis [124], a model in which enhanced levels of ROS have been demonstrated in the latent period, particularly the early one that directly follows the insult [125]. Interestingly, the anti-epileptogenic effect occurred despite that the LTCC antagonist was present only during SE (which it left unaffected). Hence, relevant effects of LTCCs may have arisen in close association with SE, either during SE, e.g., in its terminal phase, or in the immediate aftermath. Indeed, electrographic spikes were shown to occur abundantly after SE termination in this model [126]. Spike frequencies were similar to those reported for discharges in lateralized periodic discharges (LPDs), which were described to arise in the terminal phase of SE in humans [127]. Besides SE, LPDs (formerly known as PLEDs: periodic lateralized epileptiform discharges [128]) have been associated with stroke, traumatic brain injury, tumors and encephalitis, i.e., acute and chronic brain lesions widely recognized as pathologies potentially leading to epilepsy [129]. LPDs and IIS share etiological (e.g., association with SE [127,130] and morphological (interictal spike-like contributions [129]) aspects, and there are also pharmacological (resistance to anti-epileptic drug treatment [129,131]) and further pathological similarities (e.g., cognitive dysfunction [132,133]) between these two. Hence, considering the close association between LPDs and brain insult, a narrow time window for interference with potentially epileptogenic discharges [134] may also apply to the situation in humans.

Having said this, it needs to be considered that evidence arguing against a crucial role of IIS in epileptogenesis has been presented as well. The group of Staley and co-workers showed that in an organotypic hippocampal slice culture model of epileptogenesis, the occurrence of IIS indeed preceded the first appearance of ictal discharges by several days [101], and IIS/PDS could thus play a role in the processes leading to this form of “culture-dish epilepsy”. However, in a subsequent study this group demonstrated that chronic blocking of interictal discharges using the glutamate receptor antagonist kynurenic acid did not prevent the appearance of ictal discharges once it was removed [135]. One potential caveat regarding the interpretation of this finding lies in the fact that prolonged culturing of hippocampal neurons in kynurenate has been shown to lead to seizure-like activity per se upon its removal [22,29,136]. Hence, changes in excitatory neurotransmission by the chronic blockade of glutamate receptors may have replaced potential epileptogenic effects of IIS/PDS in this model.

### 4.2. An Anti-Ictogenic Role of PDS

**Figure 2:**
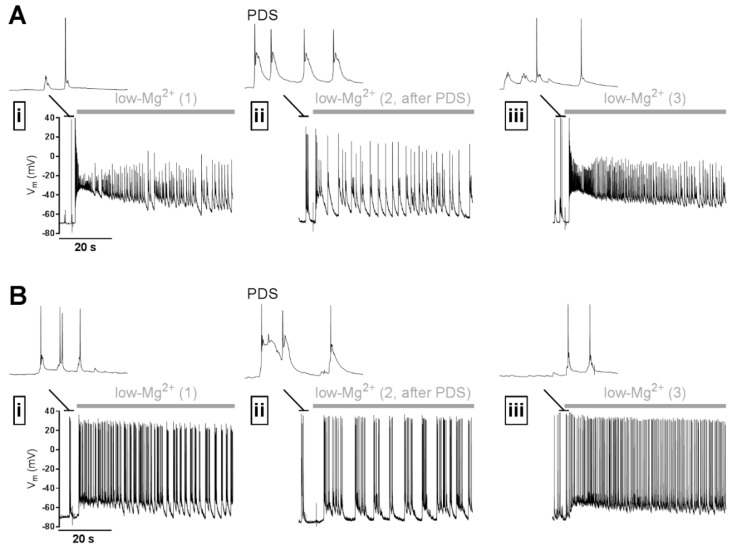
Illustration of a potential anti-ictal effect of PDS. (**A**,**B**) Two examples of electrophysiological experiments on primary rat hippocampal networks using the perforated patch-clamp technique, which indicate that PDS may inhibit seizure-like discharge activity (SLA) in an acute manner. SLA was induced repeatedly (i, ii, iii) by omission of Mg^2+^ from the superfusate (“low-Mg^2+^”-solution, application indicated by the grey horizontal bar) with recovery periods of 5 min between the stimulations. After the SLA displayed in (i), PDS were induced by co-application of 10 µM bicuculline + 3 µM Bay K8644. 5 min later, the solution was exchanged again for low Mg^2+^ solution to elicit SLA. Immediately following PDS, SLA was considerably reduced (ii). After another 5 min interval, during which PDS were not evoked, SLA appeared similar to the one during the initial control recording (iii). The discharge activity immediately preceding the switch to low-Mg^2+^ solution is shown on an expanded time scale above the SLA traces. Note that neurons showed synaptically evoked action potential discharge in (i) and (iii). Examples of the PDS that were evoked in (ii) are also displayed on the same time scale.

If it holds true that PDS show predominantly pro-epileptic effects, then it follows that a straight-forward anti-epileptogenic strategy would be to abolish IIS/PDS. However, to date there is no general agreement in the field that prevention of PDS might prove beneficial. This skepticism is primarily based on observations of an inverse relation between IIS and the likeliness of seizures, e.g., IIS frequencies remain unaltered or decrease prior to the appearance of seizures, but high frequencies were seen after the seizures [137]. Moreover, discontinuation of anti-epileptic drugs (which fail to block interictal spikes, see reference [131]) led to a decrease of interictal spike frequency prior to the increase of spontaneous seizures [138,139]. Thus, in contrast to the alternative view that an increase in IIS activity may ultimately initiate ictal discharges (interictal to ictal transition, see reference [140]), IIS may act to prevent ictal discharges. Such an inhibitory effect was indeed demonstrated in isolated brain preparations [141,142]. Moreover, interictal spikes were shown to inhibit action potential firing and seizures [85,143,144]. This effect was explained by a transient refractoriness that may be caused by the IIS/PDS in the epileptic network [88], and it was argued that IIS/PDS should therefore not be opposed in anti-epileptic therapy [86]. The question of an anti- or pro-epileptic effect of IIS/PDS has been heavily discussed for quite some time [145]. However, the potential anti-epileptic effect can be seen as an acute action of PDS on neuronal discharge activity (electrical refractoriness, which can readily be demonstrated in vitro, see Figure 2), that may operate independently of the more long-lasting pro-epileptic effects described above (illustrated in Figure 1A and in more detail in reference [55]). Hence, the controversy regarding the implication of IIS/PDS in epilepsy can possibly be attributed at least in part to different time scales in which they have been considered. On the other hand, BDNF, in addition to its potentially pro-epileptic effects described above, also displays anti-epileptic activity, an effect that appears to rely on generation of neuropeptide Y [146]. Hence, if PDS indeed lead to an elevation of BDNF, a suppressing effect of epileptiform discharges may also arise.

## 5. Potential Role in Other Neurological Diseases

PDS were initially identified in the context of epilepsy, but observations regarding their multi-unit counterpart, electrographic “interictal” spikes, indicate that they may represent pathologically relevant neuronal discharge events in various neurological diseases [133,147,148,149].

For example, interictal spikes were seen in patients with Alzheimer’s disease (AD) [150,151,152,153,154,155] and were also detected in a respective mouse model [156]. Moreover, interictal spikes occur when amyloid peptide is expressed in transgenic mice [157] (reviewed in reference [158]). Recently, evidence was provided both in an animal model and in humans that interictal spikes cause impairments in short-term memory and cognitive disruption [133,147,159]. The suppression of neuronal firing by PDS leading to intermittent neuronal dysfunction in the hippocampus was considered as a plausible mechanism [147,160]. In the developing brain, IIS may have more pronounced, long-term effects. The occurrence of IIS in frontal cortical regions was found to be linked to childhood autism, and induction of IIS in the brain of rat pups resulted in autism spectrum disorder (ASD)-like behavior (of note, gain-of-function mutations of Ca_v_1.3 channels, which provide a crucial conductance in PDS formation (see Section 2), have not only been linked to epilepsy, but were also identified in ASD patients, [76,161]). As mentioned above, PDS were suggested to represent epileptogenic events. Hence, PDS may additionally provide an attractive explanation why seizures are common in AD patients [162,163,164]. However, it remains an open question whether amyloid peptides can indeed elicit PDS, e.g., in hippocampal neurons, a brain region that is one of the earliest affected in AD [165]. Notably, membrane depolarizations that shared some (but for duration) characteristics with PDS, e.g., a pronounced dependence on AMPA receptors and modest dependence on NMDA receptors, were induced by amyloid β protofibrils and fibrils in neocortical neurons [166,167].

Furthermore, neuropathological conditions described for dopaminergic neurons involved in PD would appear prone to cause PDS formation (high reliance on LTCC activity of the innate autonomous activity of these neurons together with a predisposition towards oxidative stress as a consequence of dopamine metabolism) [168]. However, the occurrence of such events has so far not been investigated in these neurons. Hence, more research is needed to comprehensively address the potential implications of PDS in neuropathology. 

## 6. Conclusion and Outlook

Although PDS have been discovered more than 50 years ago, their potential role in neuronal diseases is only slowly obtaining broader attention. Evidently, more work is needed to establish and eventually understand in detail the implication of PDS in neuropathology. Voltage imbalances and aberrant Ca^2+^ signaling that come with PDS offer a wide range of possible cause of neuronal dysfunction. However, the acute inhibitory action on neuronal firing and potential long-term remodeling effects of PDS may also have beneficial effects. The question of why PDS actually form has not been addressed so far. We do not know whether PDS simply represent a consequence of neuronal disruption, or whether they may be part of a controlled response to avoid further damage. Clarifying these possibilities will be necessary to adequately deal with PDS occurrence in clinical therapy.

## References

[1] Matsumoto H., Ajmone Marsan C. (1964). Cortical cellular phenomena in experimental epilepsy: Interictal manifestations. Exp. Neurol..

[2] Prince D.A. (1968). The depolarization shift in “epileptic” neurons. Exp. Neurol..

[3] Johnston D., Brown T.H. (1981). Giant synaptic potential hypothesis for epileptiform activity. Science.

[4] Antoniadis A., Müller W.E., Wollert U. (1980). Inhibition of GABA and benzodiazepine receptor binding by penicillins. Neurosci. Lett..

[5] Hablitz J.J. (1984). Picrotoxin-induced epileptiform activity in hippocampus: Role of endogenous versus synaptic factors. J. Neurophysiol..

[6] Bingmann D., Speckmann E.J., Baker R.E., Ruijter J., de Jong B.M. (1988). Differential antiepileptic effects of the organic calcium antagonists verapamil and flunarizine in neurons of organotypic neocortical explants from newborn rats. Exp. Brain Res..

[7] Straub H., Speckmann E.J., Bingmann D., Walden J. (1990). Paroxysmal depolarization shifts induced by bicuculline in CA3 neurons of hippocampal slices: Suppression by the organic calcium antagonist verapamil. Neurosci. Lett..

[8] Schiller Y. (2002). Inter-ictal- and ictal-like epileptic discharges in the dendritic tree of neocortical pyramidal neurons. J. Neurophysiol..

[9] Walden J., Pockberger H., Speckmann E.J., Petsche H. (1986). Paroxysmal neuronal depolarizations in the rat motorcortex in vivo: Intracellular injection of the calcium agonist BAY K 8644. Exp. Brain Res..

[10] Witte O.W., Speckmann E.J., Walden J. (1987). Motor cortical epileptic foci in vivo: Actions of a calcium channel blocker on paroxysmal neuronal depolarizations. Electroencephalogr. Clin. Neurophysiol..

[11] Silva-Barrat C., Szente M., Menini C., Velluti J.C., Champagnat J. (2001). Muscarinic Depression of Synaptic Transmission in the Epileptogenic GABA Withdrawal Syndrome Focus. J. Neurophysiol..

[12] Sun D.A., Sombati S., DeLorenzo R.J. (2001). Glutamate Injury–Induced Epileptogenesis in Hippocampal Neurons. Stroke.

[13] Martella G., De Persis C., Bonsi P., Natoli S., Cuomo D., Bernardi G., Calabresi P., Pisani A. (2005). Inhibition of persistent sodium current fraction and voltage-gated L-type calcium current by propofol in cortical neurons: Implications for its antiepileptic activity. Epilepsia.

[14] Dreier J.P., Major S., Pannek H.-W., Woitzik J., Scheel M., Wiesenthal D., Martus P., Winkler M.K.L., Hartings J.A., Fabricius M. (2012). Spreading convulsions, spreading depolarization and epileptogenesis in human cerebral cortex. Brain.

[15] Yu W., Calos M., Pilitsis J., Shin D.S.-H. (2013). Deconstructing the neural and ionic involvement of seizure-like events in the striatal network. Neurobiol. Dis..

[16] Sanabria E.R., Su H., Yaari Y. (2001). Initiation of network bursts by Ca^2+^-dependent intrinsic bursting in the rat pilocarpine model of temporal lobe epilepsy. J. Physiol..

[17] Yaari Y., Yue C., Su H. (2007). Recruitment of apical dendritic T-type Ca^2+^ channels by backpropagating spikes underlies de novo intrinsic bursting in hippocampal epileptogenesis. J. Physiol..

[18] Becker A.J., Pitsch J., Sochivko D., Opitz T., Staniek M., Chen C.-C., Campbell K.P., Schoch S., Yaari Y., Beck H. (2008). Transcriptional upregulation of Cav3.2 mediates epileptogenesis in the pilocarpine model of epilepsy. J. Neurosci..

[19] Chen S., Su H., Yue C., Remy S., Royeck M., Sochivko D., Opitz T., Beck H., Yaari Y. (2011). An increase in persistent sodium current contributes to intrinsic neuronal bursting after status epilepticus. J. Neurophysiol..

[20] Altrup U., Wiemann M. (2003). Paroxysmal depolarization shifts (PDS) induce non-synaptic responses in neighboured neurons (buccal ganglia, Helix pomatia). Brain Res..

[21] Stanojević M., Lopicic S., Jovanovic Z., Pathak D., Pavlovic D.V., Spasic S., Nedeljkov V., Prostran M. (2015). Magnesium Effects on Nonsynaptic Epileptiform Activity in Leech Retzius Neurons. Folia Biol. (Praha).

[22] Segal M.M., Furshpan E.J. (1990). Epileptiform activity in microcultures containing small numbers of hippocampal neurons. J. Neurophysiol..

[23] Silva-Barrat C., Velluti J., Szente M., Batini C., Champagnat J. (2005). Exaggeration of epileptic-like patterns by nicotine receptor activation during the GABA withdrawal syndrome. Brain Res..

[24] Moraidis I., Bingmann D., Lehmenkühler A., Speckmann E.J. (1991). Caffeine-induced epileptic discharges in CA3 neurons of hippocampal slices of the guinea pig. Neurosci. Lett..

[25] Straub H., Köhling R., Speckmann E.J. (1994). Picrotoxin-induced epileptic activity in hippocampal and neocortical slices (guinea pig): Suppression by organic calcium channel blockers. Brain Res..

[26] Traub R.D., Miles R., Jefferys J.G. (1993). Synaptic and intrinsic conductances shape picrotoxin-induced synchronized after-discharges in the guinea-pig hippocampal slice. J. Physiol..

[27] Stiglbauer V., Hotka M., Ruiß M., Hilber K., Boehm S., Kubista H. (2017). Ca v 1.3 channels play a crucial role in the formation of paroxysmal depolarization shifts in cultured hippocampal neurons. Epilepsia.

[28] Rubi L., Schandl U., Lagler M., Geier P., Spies D., Gupta K., Boehm S., Kubista H. (2013). Raised Activity of L-Type Calcium Channels Renders Neurons Prone to Form Paroxysmal Depolarization Shifts. NeuroMolecular Med..

[29] Segal M.M. (1991). Epileptiform activity in microcultures containing one excitatory hippocampal neuron. J. Neurophysiol..

[30] Speckmann E.J., Walden J., Bingmann D. (1990). Contribution of calcium ions to epileptogenesis. J. Basic Clin. Physiol. Pharmacol..

[31] Speckmann E.J., Walden J., Schwartzkroin P.A. (1993). Anti-epileptic effects of organic calcium channel blockers. Epilepsy: Models, Mechanisms and Concepts.

[32] Baldino F., Wolfson B., Heinemann U., Gutnick M.J. (1986). An N-methyl-D-aspartate (NMDA) receptor antagonist reduces bicuculline-induced depolarization shifts in neocortical explant cultures. Neurosci. Lett..

[33] Jones R.S. (1988). Epileptiform events induced by GABA-antagonists in entorhinal cortical cells in vitro are partly mediated by N-methyl-D-aspartate receptors. Brain Res..

[34] McBain C.J., Boden P., Hill R.G. (1989). Rat hippocampal slices “in vitro” display spontaneous epileptiform activity following long-term organotypic culture. J. Neurosci. Methods.

[35] Gean P.W., Chang F.C. (1991). Ketamine suppresses synchronized discharges in the disinhibited amygdala slice. Brain Res. Bull..

[36] Hwa G.G., Avoli M., Oliver A., Villemure J.G. (1991). Bicuculline-induced epileptogenesis in the human neocortex maintained in vitro. Exp. Brain Res..

[37] Lee W.L., Hablitz J.J. (1990). Effect of APV and ketamine on epileptiform activity in the CA1 and CA3 regions of the hippocampus. Epilepsy Res..

[38] Lee W.L., Hablitz J.J. (1991). Excitatory synaptic involvement in epileptiform bursting in the immature rat neocortex. J. Neurophysiol..

[39] Akopian G., Walsh J.P. (2002). Corticostriatal paired-pulse potentiation produced by voltage-dependent activation of NMDA receptors and L-type Ca(2+) channels. J. Neurophysiol..

[40] Baginskas A., Kuras A. (2009). L-type Ca^2+^ current in frog tectal recurrent neurons determines the NMDA receptor activation on efferent neuron. Exp. Brain Res..

[41] Wang D., Grillner S., Wallén P. (2013). Calcium dynamics during NMDA-induced membrane potential oscillations in lamprey spinal neurons-contribution of L-type calcium channels (CaV1.3). J. Physiol..

[42] Fossat P., Sibon I., Le Masson G., Landry M., Nagy F. (2007). L-type calcium channels and NMDA receptors: A determinant duo for short-term nociceptive plasticity. Eur. J. Neurosci..

[43] Domann R., Westerhoff C.H., Witte O.W. (1994). Inhibitory mechanisms terminating paroxysmal depolarization shifts in hippocampal neurons of rats. Neurosci. Lett..

[44] Holmes G.L., Ben-Ari Y. (2001). The neurobiology and consequences of epilepsy in the developing brain. Pediatr. Res..

[45] Domann R., Dorn T., Witte O.W. (1991). Afterpotentials following penicillin-induced paroxysmal depolarizations in rat hippocampal CA1 pyramidal cells in vitro. Pflugers Arch..

[46] Witte O.W. (1994). Afterpotentials of penicillin-induced epileptiform neuronal discharges in the motor cortex of the rat in vivo. Epilepsy Res..

[47] Witte O.W., Uhlig S., Valle E. (1989). Separation of different types of afterpotentials following penicillin-induced paroxysmal depolarization shifts of neurons in the motor cortex of the rat. Neurosci. Lett..

[48] Westerhoff C.H., Domann R., Witte O.W. (1995). Inhibitory mechanisms in epileptiform activity induced by low magnesium. Pflugers Arch..

[49] Agrawal N., Alonso A., Ragsdale D.S. (2003). Increased persistent sodium currents in rat entorhinal cortex layer V neurons in a post-status epilepticus model of temporal lobe epilepsy. Epilepsia.

[50] Hellier J.L., Patrylo P.R., Dou P., Nett M., Rose G.M., Dudek F.E. (1999). Assessment of inhibition and epileptiform activity in the septal dentate gyrus of freely behaving rats during the first week after kainate treatment. J. Neurosci..

[51] de Curtis M., Radici C., Forti M. (1999). Cellular mechanisms underlying spontaneous interictal spikes in an acute model of focal cortical epileptogenesis. Neuroscience.

[52] Perez-Reyes E. (2003). Molecular physiology of low-voltage-activated t-type calcium channels. Physiol. Rev..

[53] Zheng F., Phelan K.D. (2014). The role of canonical transient receptor potential channels in seizure and excitotoxicity. Cells.

[54] Schiller Y. (2004). Activation of a calcium-activated cation current during epileptiform discharges and its possible role in sustaining seizure-like events in neocortical slices. J. Neurophysiol..

[55] Hotka M., Kubista H. (2018). The paroxysmal depolarization shift in epilepsy research. Int. J. Biochem. Cell Biol..

[56] Dinocourt C., Petanjek Z., Freund T.F., Ben-Ari Y., Esclapez M. (2003). Loss of interneurons innervating pyramidal cell dendrites and axon initial segments in the CA1 region of the hippocampus following pilocarpine-induced seizures. J. Comp. Neurol..

[57] Drexel M., Preidt A.P., Kirchmair E., Sperk G. (2011). Parvalbumin interneurons and calretinin fibers arising from the thalamic nucleus reuniens degenerate in the subiculum after kainic acid-induced seizures. Neuroscience.

[58] Rakhade S.N., Fitzgerald E.F., Klein P.M., Zhou C., Sun H., Huganir R.L., Hunganir R.L., Jensen F.E. (2012). Glutamate receptor 1 phosphorylation at serine 831 and 845 modulates seizure susceptibility and hippocampal hyperexcitability after early life seizures. J. Neurosci..

[59] Lopes M.W., Soares F.M.S., de Mello N., Nunes J.C., Cajado A.G., de Brito D., de Cordova F.M., da Cunha R.M.S., Walz R., Leal R.B. (2013). Time-dependent modulation of AMPA receptor phosphorylation and mRNA expression of NMDA receptors and glial glutamate transporters in the rat hippocampus and cerebral cortex in a pilocarpine model of epilepsy. Exp. Brain Res..

[60] Di Maio R., Mastroberardino P.G., Hu X., Montero L.M., Greenamyre J.T. (2013). Thiol oxidation and altered NR2B/NMDA receptor functions in in vitro and in vivo pilocarpine models: Implications for epileptogenesis. Neurobiol. Dis..

[61] Raza M., Blair R.E., Sombati S., Carter D.S., Deshpande L.S., DeLorenzo R.J. (2004). Evidence that injury-induced changes in hippocampal neuronal calcium dynamics during epileptogenesis cause acquired epilepsy. Proc. Natl. Acad. Sci. USA.

[62] DeLorenzo R.J., Sun D.A., Deshpande L.S. (2006). Erratum to “Cellular mechanisms underlying acquired epilepsy: the calcium hypothesis of the induction and maintenance of epilepsy.” [Pharmacol. Ther. 105(3) (2005) 229-266]. Pharmacol. Ther..

[63] Di Maio R., Mastroberardino P.G., Hu X., Montero L., Greenamyre J.T. (2011). Pilocapine alters NMDA receptor expression and function in hippocampal neurons: NADPH oxidase and ERK1/2 mechanisms. Neurobiol. Dis..

[64] Waldbaum S., Patel M. (2010). Mitochondrial dysfunction and oxidative stress: A contributing link to acquired epilepsy?. J. Bioenerg. Biomembr..

[65] Li X., Zhou J., Chen Z., Chen S., Zhu F., Zhou L. (2008). Long-term expressional changes of Na^+^ −K^+^ -Cl^−^ co-transporter 1 (NKCC1) and K^+^ −Cl^−^ co-transporter 2 (KCC2) in CA1 region of hippocampus following lithium-pilocarpine induced status epilepticus (PISE). Brain Res..

[66] Barmashenko G., Hefft S., Aertsen A., Kirschstein T., Köhling R. (2011). Positive shifts of the GABAA receptor reversal potential due to altered chloride homeostasis is widespread after status epilepticus. Epilepsia.

[67] Gutnick M.J., Connors B.W., Prince D.A. (1982). Mechanisms of neocortical epileptogenesis in vitro. J. Neurophysiol..

[68] Bingmann D., Speckmann E.J. (1986). Actions of pentylenetetrazol (PTZ) on CA3 neurons in hippocampal slices of guinea pigs. Exp. Brain Res..

[69] Raza M., Shaheen F., Choudhary M.I., Rahman A., Sombati S., DeLorenzo R.J. (2002). In vitro inhibition of pentylenetetrazole and bicuculline-induced epileptiform activity in rat hippocampal pyramidal neurons by aqueous fraction isolated from Delphinium denudatum. Neurosci. Lett..

[70] Akaishi T., Nakazawa K., Sato K., Saito H., Ohno Y., Ito Y. (2004). Hydrogen peroxide modulates whole cell Ca^2+^ currents through L-type channels in cultured rat dentate granule cells. Neurosci. Lett..

[71] Xu W., Lipscombe D. (2001). Neuronal Ca(V)1.3alpha(1) L-type channels activate at relatively hyperpolarized membrane potentials and are incompletely inhibited by dihydropyridines. J. Neurosci..

[72] Thomas J.R., Lee A. (2016). Measuring Ca^2+^-Dependent Modulation of Voltage-Gated Ca^2+^ Channels in HEK-293T Cells. Cold Spring Harb. Protoc..

[73] Chameau P., Qin Y., Spijker S., Smit A.B., Smit G., Joëls M. (2007). Glucocorticoids specifically enhance L-type calcium current amplitude and affect calcium channel subunit expression in the mouse hippocampus. J. Neurophysiol..

[74] Gao L., Blair L.A.C., Salinas G.D., Needleman L.A., Marshall J. (2006). Insulin-like growth factor-1 modulation of CaV1.3 calcium channels depends on Ca^2+^ release from IP3-sensitive stores and calcium/calmodulin kinase II phosphorylation of the alpha1 subunit EF hand. J. Neurosci..

[75] Klassen T., Davis C., Goldman A., Burgess D., Chen T., Wheeler D., McPherson J., Bourquin T., Lewis L., Villasana D. (2011). Exome sequencing of ion channel genes reveals complex profiles confounding personal risk assessment in epilepsy. Cell.

[76] Pinggera A., Striessnig J. (2016). Cav1.3 (CACNA1D) L-type Ca^2+^ channel dysfunction in CNS disorders. J. Physiol..

[77] De Curtis M., Jefferys J.G.R., Avoli M., Noebels J.L., Avoli M., Rogawski M.A., Olsen R.W., Delgado-Escueta A.V. (2012). Interictal Epileptiform Discharges in Partial Epilepsy Different IED Patterns in Epileptic Patients: Spikes, Spike Bursts. Jasper’s Basic Mechanisms of the Epilepsies [Internet].

[78] Jiruska P., de Curtis M., Jefferys J.G.R., Schevon C.A., Schiff S.J., Schindler K. (2013). Synchronization and desynchronization in epilepsy: Controversies and hypotheses. J. Physiol..

[79] Wong Y., Lee C., Xie W., Cui B., Poo M. (2015). Activity-dependent BDNF release via endocytic pathways is regulated by synaptotagmin-6 and complexin. Proc. Natl. Acad. Sci. USA.

[80] Kafitz K.W., Rose C.R., Thoenen H., Konnerth A. (1999). Neurotrophin-evoked rapid excitation through TrkB receptors. Nature.

[81] Sasi M., Vignoli B., Canossa M., Blum R. (2017). Neurobiology of local and intercellular BDNF signaling. Pflugers Arch..

[82] Beste C., Kolev V., Yordanova J., Domschke K., Falkenstein M., Baune B.T., Konrad C. (2010). The role of the BDNF Val66Met polymorphism for the synchronization of error-specific neural networks. J. Neurosci..

[83] Zheng K., An J.J., Yang F., Xu W., Xu Z.D., Wu J., Hökfelt T.G.M., Fisahn A., Xu B., Lu B. (2011). TrkB signaling in parvalbumin-positive interneurons is critical for gamma-band network synchronization in hippocampus. Proc. Natl. Acad. Sci. USA.

[84] Mongrain V., Warby S.C. (2012). Determinants of cortical synchrony. Sleep.

[85] Zhou J., Lenck-Santini P.-P., Zhao Q., Holmes G.L. (2007). Effect of interictal spikes on single-cell firing patterns in the hippocampus. Epilepsia.

[86] de Curtis M., Avanzini G. (2001). Interictal spikes in focal epileptogenesis. Prog. Neurobiol..

[87] Staley K., Hellier J.L., Dudek F.E. (2005). Do interictal spikes drive epileptogenesis?. Neuroscientist.

[88] Staley K.J., White A., Dudek F.E. (2011). Interictal spikes: harbingers or causes of epilepsy?. Neurosci. Lett..

[89] Ben-Ari Y., Cherubini E., Corradetti R., Gaiarsa J.L. (1989). Giant synaptic potentials in immature rat CA3 hippocampal neurones. J. Physiol..

[90] Griguoli M., Cherubini E. (2017). Early Correlated Network Activity in the Hippocampus: Its Putative Role in Shaping Neuronal Circuits. Front. Cell. Neurosci..

[91] Curia G., Longo D., Biagini G., Jones R.S.G., Avoli M. (2008). The pilocarpine model of temporal lobe epilepsy. J. Neurosci. Methods.

[92] Tyzio R., Allene C., Nardou R., Picardo M.A., Yamamoto S., Sivakumaran S., Caiati M.D., Rheims S., Minlebaev M., Milh M. (2011). Depolarizing actions of GABA in immature neurons depend neither on ketone bodies nor on pyruvate. J. Neurosci..

[93] Mohajerani M.H., Sivakumaran S., Zacchi P., Aguilera P., Cherubini E. (2007). Correlated network activity enhances synaptic efficacy via BDNF and the ERK pathway at immature CA3 CA1 connections in the hippocampus. Proc. Natl. Acad. Sci. USA.

[94] Cherubini E., Griguoli M., Safiulina V., Lagostena L. (2011). The depolarizing action of GABA controls early network activity in the developing hippocampus. Mol. Neurobiol..

[95] Deisseroth K., Mermelstein P.G., Xia H., Tsien R.W. (2003). Signaling from synapse to nucleus: the logic behind the mechanisms. Curr. Opin. Neurobiol..

[96] Ma H., Cohen S., Li B., Tsien R.W. (2012). Exploring the dominant role of Cav1 channels in signalling to the nucleus. Biosci. Rep..

[97] Hongpaisan J., Winters C.A., Andrews S.B. (2003). Calcium-dependent mitochondrial superoxide modulates nuclear CREB phosphorylation in hippocampal neurons. Mol. Cell. Neurosci..

[98] Staley K.J., Dudek F.E. (2006). Interictal spikes and epileptogenesis. Epilepsy Curr..

[99] Rakhade S.N., Shah A.K., Agarwal R., Yao B., Asano E., Loeb J.A. (2007). Activity-dependent gene expression correlates with interictal spiking in human neocortical epilepsy. Epilepsia.

[100] Wadman W.J., Da Silva F.H., Leung L.W. (1983). Two types of interictal transients of reversed polarity in rat hippocampus during kindling. Electroencephalogr. Clin. Neurophysiol..

[101] Dyhrfjeld-Johnsen J., Berdichevsky Y., Swiercz W., Sabolek H., Staley K.J. (2010). Interictal spikes precede ictal discharges in an organotypic hippocampal slice culture model of epileptogenesis. J. Clin. Neurophysiol..

[102] White A., Williams P.A., Hellier J.L., Clark S., Dudek F.E., Staley K.J. (2010). EEG spike activity precedes epilepsy after kainate-induced status epilepticus. Epilepsia.

[103] Chauvière L., Doublet T., Ghestem A., Siyoucef S.S., Wendling F., Huys R., Jirsa V., Bartolomei F., Bernard C. (2012). Changes in interictal spike features precede the onset of temporal lobe epilepsy. Ann. Neurol..

[104] Roseman E., Woodhall B. (1946). The electro-encephalogram in war wounds of the brain; with particular reference to post-traumatic epilepsy. Res. Publs. Assoc. Res. Nerv. Ment. Dis. Proc..

[105] Momiyama T., Ishihara K., Serikawa T., Moritake K., Sasa M. (1995). Effect of nicardipine on abnormal excitability of CA3 pyramidal cells in hippocampal slices of spontaneously epileptic rats. Eur. J. Pharmacol..

[106] Hanaya R., Sasa M., Kiura Y., Ishihara K., Serikawa T., Kurisu K. (2002). Epileptiform burst discharges in hippocampal CA3 neurons of young but not mature Noda epileptic rats (NER). Brain Res..

[107] McCarthy M.M., Auger A.P., Perrot-Sinal T.S. (2002). Getting excited about GABA and sex differences in the brain. Trends Neurosci..

[108] Danzer S.C., Crooks K.R.C., Lo D.C., McNamara J.O. (2002). Increased expression of brain-derived neurotrophic factor induces formation of basal dendrites and axonal branching in dentate granule cells in hippocampal explant cultures. J. Neurosci..

[109] Koyama R., Yamada M.K., Fujisawa S., Katoh-Semba R., Matsuki N., Ikegaya Y. (2004). Brain-derived neurotrophic factor induces hyperexcitable reentrant circuits in the dentate gyrus. J. Neurosci..

[110] Ikegaya Y. (1999). Abnormal targeting of developing hippocampal mossy fibers after epileptiform activities via L-type Ca^2+^ channel activation in vitro. J. Neurosci..

[111] Scharfman H., Goodman J., Macleod A., Phani S., Antonelli C., Croll S. (2005). Increased neurogenesis and the ectopic granule cells after intrahippocampal BDNF infusion in adult rats. Exp. Neurol..

[112] Sugaya Y., Maru E., Kudo K., Shibasaki T., Kato N. (2010). Levetiracetam suppresses development of spontaneous EEG seizures and aberrant neurogenesis following kainate-induced status epilepticus. Brain Res..

[113] Cameron M.C., Zhan R., Nadler J.V. (2011). Morphologic integration of hilar ectopic granule cells into dentate gyrus circuitry in the pilocarpine model of temporal lobe epilepsy. J. Comp. Neurol..

[114] Scharfman H.E., Pierce J.P. (2012). New insights into the role of hilar ectopic granule cells in the dentate gyrus based on quantitative anatomic analysis and three-dimensional reconstruction. Epilepsia.

[115] Botterill J.J., Brymer K.J., Caruncho H.J., Kalynchuk L.E. (2015). Aberrant hippocampal neurogenesis after limbic kindling: Relationship to BDNF and hippocampal-dependent memory. Epilepsy Behav..

[116] Hosford B.E., Liska J.P., Danzer S.C. (2016). Ablation of Newly Generated Hippocampal Granule Cells Has Disease-Modifying Effects in Epilepsy. J. Neurosci..

[117] Danzer S.C. (2016). Neurogenesis in Epilepsy: Better to Burn Out or Fade Away?. Epilepsy Curr..

[118] Shapiro L.A., Figueroa-Aragon S., Ribak C.E. (2007). Newly generated granule cells show rapid neuroplastic changes in the adult rat dentate gyrus during the first five days following pilocarpine-induced seizures. Eur. J. Neurosci..

[119] Hell J.W., Westenbroek R.E., Warner C., Ahlijanian M.K., Prystay W., Gilbert M.M., Snutch T.P., Catterall W.A. (1993). Identification and differential subcellular localization of the neuronal class C and class D L-type calcium channel alpha 1 subunits. J. Cell Biol..

[120] Ludwig A., Flockerzi V., Hofmann F. (1997). Regional expression and cellular localization of the alpha1 and beta subunit of high voltage-activated calcium channels in rat brain. J. Neurosci..

[121] Liebmann L., Karst H., Sidiropoulou K., van Gemert N., Meijer O.C., Poirazi P., Joëls M. (2008). Differential effects of corticosterone on the slow afterhyperpolarization in the basolateral amygdala and CA1 region: Possible role of calcium channel subunits. J. Neurophysiol..

[122] Zhang H., Fu Y., Altier C., Platzer J., Surmeier D.J., Bezprozvanny I. (2006). Ca1.2 and CaV1.3 neuronal L-type calcium channels: Differential targeting and signaling to pCREB. Eur. J. Neurosci..

[123] Xu J.H., Long L., Tang Y.C., Hu H.T., Tang F.R. (2007). Ca(v)1.2, Ca(v)1.3, and Ca(v)2.1 in the mouse hippocampus during and after pilocarpine-induced status epilepticus. Hippocampus.

[124] Mikati M.A., Holmes G.L., Werner S., Bakkar N., Carmant L., Liu Z., Stafstrom C.E. (2004). Effects of nimodipine on the behavioral sequalae of experimental status epilepticus in prepubescent rats. Epilepsy Behav..

[125] Jarrett S.G., Liang L., Hellier J.L., Staley K.J., Patel M. (2008). Mitochondrial DNA damage and impaired base excision repair during epileptogenesis. Neurobiol. Dis..

[126] Puttachary S., Sharma S., Verma S., Yang Y., Putra M., Thippeswamy A., Luo D., Thippeswamy T. (2016). 1400 W, a highly selective inducible nitric oxide synthase inhibitor is a potential disease modifier in the rat kainate model of temporal lobe epilepsy. Neurobiol. Dis..

[127] Kaplan P.W. (2007). EEG criteria for nonconvulsive status epilepticus. Epilepsia.

[128] Lin L., Drislane F.W. (2018). Lateralized Periodic Discharges: A Literature Review. J. Clin. Neurophysiol..

[129] Mader E.C., Cannizzaro L.A., Williams F.J., Lalan S., Olejniczak P.W. (2017). Periodic Lateralized Epileptiform Discharges can Survive Anesthesia and Result in Asymmetric Drug-induced Burst Suppression. Neurol. Int..

[130] Avoli M., Biagini G., de Curtis M. (2006). Do interictal spikes sustain seizures and epileptogenesis?. Epilepsy Curr..

[131] D’Antuono M., Köhling R., Ricalzone S., Gotman J., Biagini G., Avoli M. (2010). Antiepileptic drugs abolish ictal but not interictal epileptiform discharges in vitro. Epilepsia.

[132] Terzano M.G., Parrino L., Mazzucchi A., Moretti G. (1986). Confusional states with periodic lateralized epileptiform discharges (PLEDs): A peculiar epileptic syndrome in the elderly. Epilepsia.

[133] Kleen J.K., Scott R.C., Holmes G.L., Roberts D.W., Rundle M.M., Testorf M., Lenck-Santini P.-P., Jobst B.C. (2013). Hippocampal interictal epileptiform activity disrupts cognition in humans. Neurology.

[134] Andraus M.E.C., Andraus C.F., Alves-Leon S.V. (2012). Periodic EEG patterns: Importance of their recognition and clinical significance. Arq. Neuropsiquiatr..

[135] Berdichevsky Y., Dzhala V., Mail M., Staley K.J. (2012). Interictal spikes, seizures and ictal cell death are not necessary for post-traumatic epileptogenesis in vitro. Neurobiol. Dis..

[136] Segal M.M. (1994). Endogenous bursts underlie seizurelike activity in solitary excitatory hippocampal neurons in microcultures. J. Neurophysiol..

[137] Gotman J. (1991). Relationships between interictal spiking and seizures: human and experimental evidence. Can. J. Neurol. Sci..

[138] Spencer S.S., Goncharova I.I., Duckrow R.B., Novotny E.J., Zaveri H.P. (2008). Interictal spikes on intracranial recording: Behavior, physiology, and implications. Epilepsia.

[139] Goncharova I.I., Alkawadri R., Gaspard N., Duckrow R.B., Spencer D.D., Hirsch L.J., Spencer S.S., Zaveri H.P. (2016). Clinical Neurophysiology The relationship between seizures, interictal spikes and antiepileptic drugs. Clin. Neurophysiol..

[140] Avoli M. (2001). Do interictal discharges promote or control seizures? Experimental evidence from an in vitro model of epileptiform discharge. Epilepsia.

[141] Barbarosie M., Avoli M. (1997). CA3-driven hippocampal-entorhinal loop controls rather than sustains in vitro limbic seizures. J. Neurosci..

[142] Librizzi L., de Curtis M. (2003). Epileptiform ictal discharges are prevented by periodic interictal spiking in the olfactory cortex. Ann. Neurol..

[143] de Curtis M., Librizzi L., Biella G. (2001). Discharge threshold is enhanced for several seconds after a single interictal spike in a model of focal epileptogenesis. Eur. J. Neurosci..

[144] Yan W., Mitzelfelt J.D., Principe J.C., Sanchez J.C. (2008). The effects of interictal spikes on single neuron firing patterns in the hippocampus during the development of temporal lobe epilepsy. Conf. Proc. Annu. Int. Conf. IEEE Eng. Med. Biol. Soc. IEEE Eng. Med. Biol. Soc. Annu. Conf..

[145] Rogawski M.A. (2006). Point-counterpoint: Do interictal spikes trigger seizures or protect against them?. Epilepsy Curr..

[146] Reibel S., Vivien-Roels B., Lê B.T., Larmet Y., Carnahan J., Marescaux C., Depaulis A. (2000). Overexpression of neuropeptide Y induced by brain-derived neurotrophic factor in the rat hippocampus is long lasting. Eur. J. Neurosci..

[147] Kleen J.K., Scott R.C., Holmes G.L., Lenck-Santini P.P. (2010). Hippocampal interictal spikes disrupt cognition in rats. Ann. Neurol..

[148] Pignatelli M., Lebreton F., Cho Y.H., Leinekugel X. (2012). “Ectopic” theta oscillations and interictal activity during slow-wave state in the R6/1 mouse model of Huntington’s disease. Neurobiol. Dis..

[149] Kam K., Duffy Á.M., Moretto J., LaFrancois J.J., Scharfman H.E. (2016). Interictal spikes during sleep are an early defect in the Tg2576 mouse model of β-amyloid neuropathology. Sci. Rep..

[150] Foncin J.F., Salmon D., Supino-Viterbo V., Feldman R.G., Macchi G., Mariotti P., Scoppetta C., Caruso G., Bruni A.C. (1985). Alzheimer’s presenile dementia transmitted in an extended kindred. Rev. Neurol. (Paris).

[151] Ito M., Echizenya N., Nemoto D., Kase M. (2009). A case series of epilepsy-derived memory impairment resembling Alzheimer disease. Alzheimer Dis. Assoc. Disord..

[152] Vossel K.A., Beagle A.J., Rabinovici G.D., Shu H., Lee S.E., Naasan G., Hegde M., Cornes S.B., Henry M.L., Nelson A.B. (2013). Seizures and epileptiform activity in the early stages of Alzheimer disease. JAMA Neurol..

[153] Vossel K.A., Ranasinghe K.G., Beagle A.J., Mizuiri D., Honma S.M., Dowling A.F., Darwish S.M., Van Berlo V., Barnes D.E., Mantle M. (2016). Incidence and impact of subclinical epileptiform activity in Alzheimer’s disease. Ann. Neurol..

[154] Lam A.D., Deck G., Goldman A., Eskandar E.N., Noebels J., Cole A.J. (2017). Silent hippocampal seizures and spikes identified by foramen ovale electrodes in Alzheimer’s disease. Nat. Med..

[155] Vossel K.A., Tartaglia M.C., Nygaard H.B., Zeman A.Z., Miller B.L. (2017). Epileptic activity in Alzheimer’s disease: Causes and clinical relevance. Lancet. Neurol..

[156] Reyes-Marin K.E., Nuñez A. (2017). Seizure susceptibility in the APP/PS1 mouse model of Alzheimer’s disease and relationship with amyloid β plaques. Brain Res..

[157] Palop J.J., Mucke L. (2009). Epilepsy and cognitive impairments in Alzheimer disease. Arch. Neurol..

[158] Noebels J. (2011). A perfect storm: Converging paths of epilepsy and Alzheimer’s dementia intersect in the hippocampal formation. Epilepsia.

[159] Binnie C.D. (2003). Cognitive impairment during epileptiform discharges: Is it ever justifiable to treat the EEG?. Lancet Neurol..

[160] Holmes G.L. (2014). What is more harmful, seizures or epileptic EEG abnormalities? Is there any clinical data?. Epileptic Disord..

[161] Pinggera A., Mackenroth L., Rump A., Schallner J., Beleggia F., Wollnik B., Striessnig J. (2017). New gain-of-function mutation shows CACNA1D as recurrently mutated gene in autism spectrum disorders and epilepsy. Hum. Mol. Genet..

[162] Gleichmann M., Chow V.W., Mattson M.P. (2011). Homeostatic disinhibition in the aging brain and Alzheimer’s disease. J. Alzheimers. Dis..

[163] Larner A.J., Marson A.G. (2011). Epileptic seizures in Alzheimer’s disease: Another fine MESS?. J. Alzheimers. Dis..

[164] Wang Y., Mattson M.P. (2014). L-type Ca^2+^ currents at CA1 synapses, but not CA3 or dentate granule neuron synapses, are increased in 3xTgAD mice in an age-dependent manner. Neurobiol. Aging.

[165] Mu Y., Gage F.H. (2011). Adult hippocampal neurogenesis and its role in Alzheimer’s disease. Mol. Neurodegener..

[166] Hartley D.M., Walsh D.M., Ye C.P., Diehl T., Vasquez S., Vassilev P.M., Teplow D.B., Selkoe D.J. (1999). Protofibrillar intermediates of amyloid beta-protein induce acute electrophysiological changes and progressive neurotoxicity in cortical neurons. J. Neurosci..

[167] Ye C., Walsh D.M., Selkoe D.J., Hartley D.M. (2004). Amyloid beta-protein induced electrophysiological changes are dependent on aggregation state: N-methyl-D-aspartate (NMDA) versus non-NMDA receptor/channel activation. Neurosci. Lett..

[168] Surmeier D.J., Schumacker P.T., Guzman J.D., Ilijic E., Yang B., Zampese E. (2017). Calcium and Parkinson’s disease. Biochem. Biophys. Res. Commun..

